# British Medical Association

**Published:** 1901-08-03

**Authors:** 


### HOSPITAL CLINICS AND MEDICAL PROGRESS.

### BRITISH MEDICAL ASSOCIATION.

The annual meeting of the British Medical Association, which this year takes place at Cheltenham, was opened on Monday evening by an "At Home" given by the Mayor and Mayoress at the Annual Exhibition and Museum which is being held in the Winter Garden. Besides the English members, a considerable number of visitors are attending the meeting, among whom are Professor Dock, of Michigan; Professor Fischer; Dr. Sabouraud, Paris; Sir William Hingston, Montreal; Professor Osler, Philadelphia; Dr. Doyen, Paris; and several colonial delegates. On Tuesday the proceedings commenced with a service at St. Matthew's Church, conducted by the rector and attended by the Mayor and Corporation, at which many of the members attending wore academic dress. The sermon was preached by the Dean of Gloucester. At the afternoon meeting Dr. Elliston, the retiring President, before giving up the chair gave an account of the progress of the Association during the year, and in the evening Dr. Ferguson, the new president, delivered his address. The discussion on the report of the constitution committee is, of course, the main subject of interest outside scientific matters, but with that we will deal later.

